# Understanding the role of digital immersive technology in educating the students of english language: does it promote critical thinking and self-directed learning for achieving sustainability in education with the help of teamwork?

**DOI:** 10.1186/s40359-024-01636-6

**Published:** 2024-03-13

**Authors:** Fenghua Tang

**Affiliations:** School of Foreign Studies, Hubei Normal University, 435000 Huangshi, China

**Keywords:** Digital immersive technology, Sustainable education, Critical thinking, Teamwork, Self-directed learning

## Abstract

**Purpose:**

Universities are constantly searching for best practices to promote sustainability when it comes to educating students of the English language. Although this area of study has recently gained the attention of scholars around the world there is still a need to explore it from various perspectives.

**Objective:**

This study aims to comprehensively investigate the impact of digital immersive technology on the education of English language students, specifically focusing on its potential to promote critical thinking and self-directed learning for achieving sustainability in education through teamwork. The research will assess the influence of digital immersive experiences on enhancing learning outcomes, examining their role in fostering critical thinking skills and encouraging self-directed learning practices. Additionally, the study explores the collaborative aspects of digital immersive technology, evaluating its contribution to teamwork among students.

**Methodology:**

The objective was achieved by using a survey questionnaire to collect data from 304 registered students in various universities in Beijing. Data analysis was conducted by applying Mplus 7.0 software.

**Findings:**

The findings revealed that the use of digital immersive technology was pivotal for achieving sustainable education both directly and indirectly to an extent. In addition, team working moderated all the respective paths except the path involving the use of digital immersive technology and critical thinking.

**Implications:**

These results generated implications for teachers and policymakers to promote and facilitate the use of digital technology for teaching the English language to students, encouraging them to develop critical skills and self-directed learning strategies. The study also offered guidance and deeper understanding for researchers to address the concerns linked to the use of digital technology and sustainable education particularly in their future endeavors.

**Supplementary Information:**

The online version contains supplementary material available at 10.1186/s40359-024-01636-6.

## Introduction

English language training has become increasingly important in the globalized world, where it serves as the primary language for communication in various fields [1]. However, conventional teaching methods may not be sufficient to equip students with the critical thinking and independent learning skills necessary to effectively apply the English language in real-life situations and address sustainability challenges [2]. As such, it is essential to investigate the role of immersive digital technology in promoting critical thinking and independent learning in English language education for achieving sustainability and how collaboration can enhance the effectiveness of this approach.

Constructivism serves as one theoretical foundation for using digital immersive technologies in education, as it posits that students actively construct their understanding of the world through experiences and interactions with their environment [3]. This process can aid digital immersive technology by providing students with access to interactive, realistic worlds to explore and experiment with, allowing them to gain a deeper understanding of concepts and ideas.

Digital immersive technology can encourage English language students to actively engage with the language, explore its many facets, and try various techniques to foster critical thinking, self-directed learning, and teamwork [4–7]. It may result in students having more confidence in their skills and better language comprehension. Digital immersive technology can also assist students in identifying areas for improvement by providing them with immediate feedback on their performance. It can help students develop their critical thinking and self-directed learning abilities. Students can build cooperation and communication skills through peer collaboration, allowing them to succeed in their studies and future employment [5].

English language instruction is essential for developing a learner’s linguistic abilities, cultural sensitivity, and sense of civic responsibility. Conventional teaching techniques, including lectures and rote memorization, might not be enough to allow students to utilize English successfully in everyday circumstances and deal with sustainability-related issues [6]. Thus, there is a rising interest in examining how teamwork may support this process and the function of digital immersion technology in fostering critical thinking and self-directed learning for achieving sustainability in English language teaching [7]. In the context of this study, the concept of teamwork is intricately woven into the broader framework of digital immersive technology’s influence on the education of English language students. Teamwork is examined as a collaborative element facilitated by digital immersive experiences, wherein students engage in joint learning activities, projects, or simulations. The relationship between teamwork and the other variables—critical thinking, self-directed learning, and sustainability—is crucial for understanding the holistic impact of digital immersive technology in education.

Teamwork is anticipated to play a pivotal role in fostering critical thinking skills among students. Through collaborative efforts, students are likely to engage in diverse perspectives, problem-solving scenarios, and analytical discussions, thereby contributing to the development of critical thinking abilities. Additionally, the collaborative nature of teamwork is expected to enhance self-directed learning practices by encouraging students to take ownership of their learning within a group dynamic. The exchange of ideas, shared responsibilities, and peer-to-peer learning within a team may empower students to autonomously direct their educational journeys [2.3,5].

Moreover, the collaborative environment fostered by teamwork is envisaged to have positive implications for sustainability in education. By working together towards common goals, students may develop a sense of shared responsibility for their learning outcomes, creating a sustainable learning community. This communal approach is likely to contribute to the longevity and effectiveness of educational practices, reinforcing the notion of sustainability within the broader educational context [6].

In essence, the relationship between teamwork and critical thinking, self-directed learning, and sustainability represents a symbiotic dynamic, wherein the collaborative nature of teamwork is anticipated to positively influence each of these variables, ultimately shaping a more comprehensive and effective educational experience for English language students within the realm of digital immersive technology.

This article aims to examine the contribution of digital immersion technology to the promotion of critical thinking and self-directed learning, as well as how teamwork might increase the efficacy of this method. The problem statement in the report goes on to describe the study’s goals and research questions. An overview of the research strategy and methodologies provide as the publication ends.

Virtual and augmented reality are two forms of immersive digital technology that increasingly use in various educational contexts, including English language instruction. Digital immersive technology offers students an immersive and interactive learning environment that can boost motivation, engagement, and memory recall [8]. Digital immersive technology can use in English language instruction to create virtual environments that mimic real-life circumstances, where students can practice their language skills, gain confidence, and increase their cultural understanding [9]. Digital immersive technology can provide learners with immediate feedback, personalized learning paths, and collaborative learning opportunities. Critical thinking is an essential skill that enables learners to analyze, evaluate, synthesize information, and make informed decisions and judgments [10]. Necessary thinking abilities are crucial for learning English since it requires students to comprehend and evaluate complicated texts and successfully communicate their views in a multilingual and multicultural setting [11]. Digital immersive technology can encourage critical thinking by allowing students to practice problem-solving, making decisions, and reflecting [12]. For instance, students can investigate other viewpoints and cultural contexts, assess the reliability of sources, and create their arguments and conclusions using immersive digital technology [13]. Another crucial ability is self-directed learning, which empowers students to take charge of their education, establish their objectives, and track their advancement. Self-directed learning is especially vital in English language education, where students must develop their language skills across various subjects [14]. For example, learners can use immersive digital technology to access authentic materials, practice their language skills in different contexts and situations, and receive personalized feedback and support. Digital immersion technology promotes critical thinking and self-directed learning in English language education for sustainability, and teamwork is another crucial component. Collaboration, communication, and social learning can all facilitate by teamwork [15]. Also, teamwork may give students a variety of perspectives, abilities, and experiences, as well as the ability to solve challenging challenges and accomplish shared objectives. Teamwork can utilizes in teaching the English language to foster collaborative learning settings for students [16].

The current study aims to explore the role of immersive digital technology in instructing English language learners and its potential to foster critical thinking and self-directed learning. The relationship between the usage of immersive digital technology and critical thinking and self-directed learning will examine, as well as the moderating impact of teamwork. The importance of this study rests in the fact that it will add to the body of knowledge already available on the use of technology in language instruction and give insight into how digital immersive technology may use to support sustainable education. To achieve sustainability in education, the results of this study will be helpful to language educators and policymakers in developing methods for integrating digital immersion technology in language teaching and learning.

The English language is widely used in international commerce, academia, and the media and has emerged as a lingua franca on a global scale. As a result, learning is essential for students who want to flourish in the worldwide economy. Additionally, one of the most often used methods of language instruction worldwide is teaching English. The demand for creative language teaching strategies that can encourage critical thinking, self-directed learning, and teamwork among English language learners, this study focuses on English language teaching. Even though the study’s primary focus is on teaching English, its findings might also apply to other languages [17]. Many of the guiding principles of digital immersive technology, such as stimulating inquiry, giving prompt feedback, and encouraging collaboration, probably apply to various languages [17]. In order to obtain the best outcomes, digital immersive technology may need to tailor to each language’s features and challenges specifically. Therefore, this study’s results may be insightful into language teaching methods, but they might not be directly applicable to other languages without additional study and adaption.

Although there is a growing corpus of studies on technology’s usage in language learning, there is a shortage of studies on how digital immersive technology might foster self-directed learning and critical thinking to achieve sustainability in education. There has been little research on immersive digital technology like virtual and augmented reality, with most studies concentrating on traditional technology like computers and multimedia in language teaching and learning. Also, as it does not cover in the existing research, it is necessary to investigate the potential moderating impact of teamwork on the relationship between the use of immersive digital technology and critical thinking and self-directed learning. By examining the potential of immersive digital technology to advance sustainable education in language teaching and learning, this study seeks to fill these gaps in the literature.

The study will be divided into several components to accomplish its goals. An overview of the body of research on digital immersion technology, critical thinking, self-directed learning, teamwork, and English language instruction will cover in the first portion. This section will present the study’s theoretical foundation and point out any gaps in the knowledge that require filling. The second section will involve creating and distributing a survey questionnaire to English language instructors and students to learn more about how they use digital immersive technology. The third portion will deal with selecting and using specific digital immersion technologies in teaching the English language, emphasizing those successful in the prior study. Additionally, information on students’ linguistic abilities, motivation, and involvement in the learning process will gather in this part, along with information on their critical thinking, self-directed learning, and teamwork abilities. The analysis and interpretation of the data will cover in the last portion, which will combine quantitative and qualitative techniques. The study’s findings will apply to create best practices for integrating digital immersion technologies into English language instruction and pinpoint areas requiring more study.

## Literature review

The writing on the expected benefits of immersive digital technology for supportable instruction is extending. A few critical ends from report surveys are recorded below: With the making of intuitive and vivid growth opportunities that empower understudies to investigate complex ideas in additional significant ways, computerized drenching innovations, like virtual and expanded reality, can help understudy commitment in finding out about maintainability [18].Digital immersive technology can give a more exhaustive comprehension of maintainability ideas by permitting students to collaborate with and picture complex information and frameworks and experience the results of ecological choices [19].Digital immersive technology can assist with advancing uplifting outlooks and ways of behaving towards maintainability by giving students a more private and profound association with natural issues [20]. Digital immersive technology can expand the availability of economic training by furnishing students with remote admittance to instructive assets and decreasing the expense of feasible schooling [21]. Using immersive digital technology in supportable training can upgrade commitment, figuring out mentalities, conduct, and openness.

### H1:

Use of digital immersive technology will positively influence sustainable education.

Immersive technology, like virtual and expanded reality, can increment understudy commitment and inspiration in picking up and improving strong reasoning abilities. Immersive technology permits students to picture and associate with complex ideas and information in manners that conventional techniques don’t allow. It can assist students with creating strong reasoning abilities by empowering them to distinguish examples and connections that are not quickly evident [22]. Immersive technology can reproduce certifiable issues and situations, permitting students to foster critical thinking abilities through experimentation, trial, and error, and coordinated effort [6]. Immersive technology can reproduce certifiable issues and situations, permitting students to foster critical thinking abilities through experimentation, trial, and error, and coordinated effort [23]. Immersive technology can reproduce cooperative workplaces, which can help students.

By and large, Immersive technology can upgrade strong reasoning abilities by expanding commitment, giving open doors to perception, further developing critical thinking, working with reflection, and advancing collaboration. Notwithstanding, it is crucial to note that different variables, including the nature of the innovation and the informative plan of the instructive experience, impact the viability of vivid innovation on decisive reasoning.

### H2:

Use of digital immersive technology will be positively linked to critical thinking.

The utilization of immersive technology can affect independent learning in different ways decidedly. The following are critical discoveries from writing surveys: immersive technology can expand students’ inspiration and commitment to training, upgrading independent knowledge. By furnishing students with an intuitive and drawing-in learning climate, immersive technology can urge them to control their insight more [24]. Immersive technology can use to make customized opportunities for growth that address individual students’ issues and inclinations. Immersive technology can uphold independent advancement by furnishing students with decisions and command over their growth opportunities [25].Immersive technology can give understudies versatility as to when and where they learn. Understudies can get Immersive technology learning experiences at whatever point and from any area, supporting free headway by engaging them to control their learning plan [26].

Immersive technology can outfit understudies with experiential learning and essential entryways that help autonomous learning. By imitating authentic circumstances and experiences, Vivid innovation can engage understudies to research and learn through trial and error 27].Immersive technology can work with reflection and self-assessment, fundamental pieces of autonomous learning. By outfitting understudies with significant opportunities to overview and consider their learning experiences, Immersive technology can maintain understudies to characterize their goals and evaluate their progression [28].

By and large, innovation can maintain autonomous headway by extending motivation and responsibility, engaging redid getting the hang of, giving flexibility, working with experiential learning, and supporting reflection and self-assessment. Regardless, the ampleness of immersive technology on free learning by various factors, including the idea of the advancement and the educational arrangement of the informational experience.

### H3:

Use of digital immersive technology positively will influence the self-directed learning.

The audit observed that decisive reasoning is a fundamental part of maintainability schooling. It empowers students to comprehend complex supportability issues. Another efficient survey analyzed the effect of powerful reasoning on manageability schooling in advanced education. The investigation discovered that decisive reasoning upgrades understudies’ capacity to break down and assess supportability issues and advances their commitment to economic practices [29].A composing overview focused on the joining of definitive thinking in natural tutoring. The examination found that thinking skills are significant for ecological training, enabling understudies to sort out the complexity of typical issues and cultivate practical plans. The review saw that unequivocal thinking is an essential capacity for teachers preparing projects to elevate their students to propel affordable tutoring [30].The analyst found the impact of innovation upheld learning conditions on decisive reasoning and maintainability training. The examination found that innovation-upheld learning conditions can improve essential reasoning abilities and advance maintainability instruction [31].

These new composing studies propose unequivocal thinking as a focal piece of functional preparation. It engages understudies to appreciate and analyze complex practical issues, cultivate rational plans, and partake in acceptable practices. This way, arranging traditional thinking skills into sensibility, preparing projects, and encouraging effective strategies to progress unequivocal thinking in understudies is essential.

### H4:

Critical thinking will positively predict sustainable educatio.

Self-directed learning is a cycle wherein understudies get to business and plan, coordinate, and evaluate their valuable learning experiences. A couple of assessments have shown the way that free learning can firmly impact efficient tutoring. For example, A review found that free learning can deal with students’ obligation to legitimacy-related activities and advance perception so they could decipher sensibility issues [32].Another review analyzed the impact of an independent learning mediation on the maintainability of schooling in advanced education [33, 34].The investigation discovered that independent learning worked on understudies’ inspiration and commitment to the framework, empowering them to foster more compelling answers for supportability challenges [35].A survey analyzed the effect of independent learning on maintainability capabilities in educator schooling [36].

These investigations propose that independent learning can affect economic training by working on students’ commitment, understanding, and use of supportability standards. Hence, integrating independent learning techniques into supportability instruction projects can be a powerful method for advancing economic training.

### H5:

Self-directed learning will positively impact the sustainable education.

Here is a composing overview of the mediating position of unequivocal thinking in the association between the use of distinctive mechanized development and practical preparation: The examination found that fundamental thinking skills mediated the association between the usage of increased reality and understudies’ obligation to legitimacy-related works out [37]. Another overview analyzed the effect of immersive digital technology on manageable training in advanced education. The investigation discovered that strong reasoning abilities intervened in the connection between using advanced digital immersive technology and students’ commitment to maintainability-related exercises [6].A survey investigated involving immersive digital technology in supportability schooling. The investigation discovered that strong reasoning abilities could improve through advanced immersive digital technology, which can advance comprehension students might interpret supportability issues and their capacity to apply manageability standards in certifiable settings [38]. A review researched the impact of a versatile application on strong reasoning abilities in maintainability schooling. The investigation discovered that essential reasoning abilities interceded the connection between portable application use and students’ commitment to manageability-related exercises [39].

Generally speaking, these investigations recommend that strong reasoning abilities can intervene in the connection between the utilization of immersive digital technology and maintainable schooling. This way, integrating techniques that move strong reasoning abilities into digital immersive technology-based supportability training projects can advance feasible education.

### H6:

Critical thinking plays a mediating role in the relationship between the use of digital immersive technology and sustainable education.

A review explored the connection between immersive digital technology and supportable training in advanced education. The investigation discovered that independent learning intervenes in this relationship, upgrading students’ inspiration and commitment to economic instruction [33]. Another overview analyzed the impact of a virtual reality-based put-together learning climate concerning manageable training in optional schools. The investigation discovered that independent learning interceded the connection between utilizing virtual reality-based reality and students’ commitment to manageability-related exercises [28].A survey investigated the utilization of expanded reality in manageability schooling. The survey found that independent learning can upgrade students’ commitment and inspiration in extended reality-based manageability schooling [27]. A review examined the impact of a portable application on practical instruction in advanced education. The investigation discovered that independent learning interceded the connection between mobile application utilization and students’ commitment to maintainability-related exercises [40].

These investigations recommend that independent learning can connect immersive digital technology and supportable training. By advancing students’ inspiration and commitment, independent learning can upgrade the viability of immersive digital technology in offering practical training. In this manner, coordinating independent learning techniques into immersive digital technology supportability training projects can be a compelling method for increasing manageable schooling.

### H7:

Self-directed learning will mediate the relationship of digital immersive technology and sustainable education.

A writing survey on the directing job of the group working in the connection between the utilization of computerized vivid innovation and feasible training: A review analyzed the effect of a virtual reality-based learning experience put together learning climate concerning supportable schooling in advanced education. The investigation discovered that group work directed the connection between the utilization of computer-generated reality and students’ commitment to maintainability-related exercises. In particular, students who worked in bunches were more committed to maintainability-related practices than those who worked exclusively [38]. The investigation discovered that group work connected learning and students’ commitment to supportability-related activities. In particular, students who worked in bunches exhibited more significant levels of responsibility in supportability-related practices than those who worked exclusively [41].A survey investigated involving computerized vivid innovation in maintainability training. The survey found that group work can be a primary consideration in advancing students’ commitment and inspiration in supportability-related exercises while utilizing immersive digital technology [42].A review researched the impact of a portable application on supportable training in advanced education. The investigation discovered that group work directed the connection between versatile application use and students’ commitment to manageability-related exercises. In particular, students who worked in bunches showed more significant levels of responsibility in supportability-related practices than those who worked separately [43].

These examinations propose that group work can connect immersive digital technology and maintainable training. By advancing cooperation and communication among students, group working can upgrade the viability of immersive digital technology in offering economic schooling. Along these lines, integrating group working systems into advanced immersive technology-based manageability training projects can move financial instruction.

### H8:

Team working will positively moderate the relationship of the use of digital immersive technology and sustainable education.

A literature review on the fortifying job of the group working in the connection between the utilization of immersive digital technology and decisive reasoning: A study analyzed the impact of a cooperative computer-generated simulation mastering climate on essential reasoning abilities in advanced education. The investigation discovered that group work fortified the connection between computer-generated reality and powerful reasoning abilities. In particular, students who worked in bunches showed more critical upgrades in vital reasoning abilities than those who worked separately [44].Another review explored the effect of a gamified mastering climate on strong reasoning abilities in essential schooling. The investigation discovered that group work fortified the connection between gamified mastering and basic reasoning abilities. In particular, students who worked in bunches exhibited more huge enhancements in strong reasoning abilities than those who worked separately [45].A survey investigated the utilization of computerized vivid innovation in advancing vital reasoning abilities in training. The survey found that group work can urgently upgrade students’ strong reasoning abilities while utilizing immersive digital technology [23]. A review examined a portable application’s impact on advanced education’s critical reasoning abilities. The investigation discovered that group work reinforced the connection between versatile application use and powerful reasoning abilities. In particular, students who worked in bunches showed more critical upgrades in essential reasoning abilities than those who worked exclusively [46].

These examinations recommend that group work can fortify the connection between immersive digital technology and powerful reasoning abilities. By advancing coordinated effort and communication among students, group work can improve the adequacy of immersive digital technology in encouraging critical reasoning abilities. Consequently, integrating group working methodologies into digital immersive technology schooling projects can advance decisive reasoning and upgrade students’ ability to apply powerful logic in certifiable settings.

### H9:

Team working strengthens the relationship of the use of digital immersive technology and critical thinking.

A review researched the impact of a cooperative computer-generated simulation learning climate on self-directed learning in advanced education. The investigation discovered that group work connected computer-generated reality and self-directed learning. In particular, students who worked in bunches showed a vast improvement in independent learning than those who worked individually [24].Another review analyzed the effect of a gamified learning climate on independent education in elementary schools. The investigation discovered that group work connected gamified learning and independent learning. In particular, students who worked in bunches exhibited more significant levels of independent education than those who worked exclusively [47].A survey investigated the utilization of immersive digital technology in advancing independent learning in training. The survey found that group work can be a basic calculation improving students’ independent realizing while utilizing immersive digital technology [24]. A review explored the impact of a portable application on independent learning in advanced education. The investigation discovered that group work connected mobile application use and independent learning. In particular, students who worked in bunches showed more improvement in independent learning than those who worked separately [45].

**Fig. 1:**
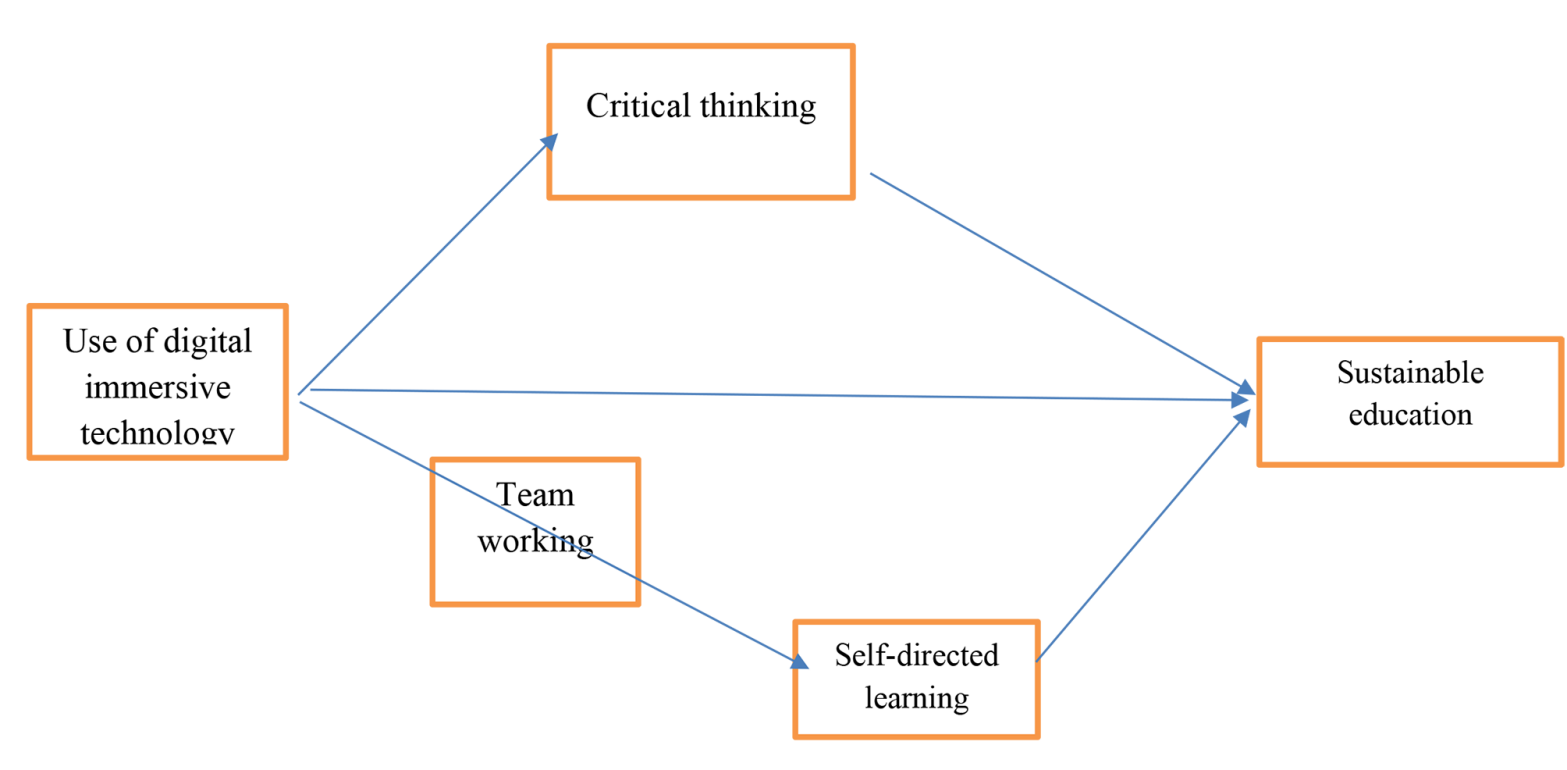
Theoretical framework

These examinations propose that group work can connect immersive digital technology and independent learning. By advancing coordinated effort among students, group working can upgrade the viability of promoting independent education. Consequently, integrating group working techniques into advanced digital immersive technology training projects can be a powerful method for empowering independent learning and upgrading students’ capacity to take responsibility for a growing experience. The relationship between the variables can be seen in Fig. 1.

Based on Fig. 1, and in line with above mentioned literature, the following hypothesis is stated:

### H10:

Team working will moderate the association of the use of digital immersive technology and self-directed learning.

## Research methodology

### Population, sampling and data collection

This research utilized the foundations of quantitative research design for hypothesis testing [48].The target population of the study consisted of the students of various universities located in Beijing. The reason to select the target population from the universities located in Beijing is that, the city is ranked no. 1 when it comes to higher education institutes [49]. This can better represent the other regions as well and the results obtained from this city will be generalizable too. For selecting the respondents form the target population, we visited the universities physically to contact with the respondents’ and to explain the purpose of the study. Students were asked various preliminary questions about the use of technology in their education and only those were selected who tend to use or have used digital technology to leverage their studies. We thus applied conveniently and purposefully selected the respondents [50]; non-probability sampling techniques, which were helpful to select the respondents according to the objectives of the study. Moreover, we first approached the student’s help desk, cafeterias to easily access the respondents. The students who were willing to participate were explained the purpose and scope of the study. After this, a standardized survey questionnaire was shared with the respondents to get their responses. The first section of the questionnaire asked some basic questions i.e., degree program, field of study, level of degree, gender, access to internet, etc., The second section comprised of questions related to the key variables of the study. A total of 304 respondents filled the questionnaire out of the distributed questionnaires which were used for further analysis.

### Data collection

This research was composed of 5 different variables: an independent variable “use of digital immersive technology”, a dependent variable “sustainable education”, two mediating variables “critical thinking” and “self-directed learning” and a moderating variable (team working). The research instrument was based upon these 5 variables and contained 27 questions specifically related to these 5 variables. The data was collected about these questions on a seven-point Likert scale ranging from 1 to 7 (1 = Strongly Disagree to 7 = Strongly Agree). All the questions were adapted from previous researches and were already validated. To meet the objectives of current study, we modified 5 items of the variable “use of digital immersive technology”, 3 items of sustainable education [51], 5 items of critical thinking [52], 5 items of self-directed learning [53] and 9 items team working [54] (See Appendix).

### Data analysis

In order to meet the objectives of the study, we conducted data analysis by utilizing the valid responses from 304 respondents. Notably, the study composed of 5 variables i.e., use of digital immersive technology, critical thinking, self-directed learning, sustainable education, and team working. It was examined whether direct and indirect association exists between the variables of the study and whether these relationships were subject to the variation of the moderating variable “team working”. For this purpose, we applied SEM approach by employing Mplus [55]. But before moving to the hypothesis testing, various preliminary assessments were made including “multivariate normality testing”, “model fitness testing through confirmatory factor analysis” and the “test of common method bias”. Mplus 7 requires the shorter names of the variables to be assigned for their effective handling. Therefore, for conveniently operating the software for data analysis, it is recommended that the names of the variables should not exceed 8 characters [56] and 90 characters per line [57]. Keeping this in mind, we shortened the names of study variables i.e., use of digital immersive technology, sustainable education, critical thinking, self-directed learning and team working to “DGIT”, “SED”, “CRT”, “SDL” and “Team”, respectively.

## Findings

### Model fitness

Prior to the assessment of hypothesis, mode-fit indices were examined to find out whether the data optimally fits with the conceptual model. It was essential to conduct the confirmatory factor analysis as the aim of the study was to test the theory. We therefore, the examined the famous model fit indices generated through Mplus i.e., chi-square, SRMR, RMSEA, CFI, and TLI, and their values were compared with the cut-off criteria. All the models fit-indices reflected that the model is perfectly fit for hypothesis testing. The details of the model fit indices are provided in Table 1.

**Table 1:** Model fitness

Measurement Model	X^2^	DF	X^2^/DF	CFI	TLI	RMSEA	SRMR
1	592.142	313	1.891	0.950	0.944	0.054	0.053

*Noten* = 304, X^**2**^ = Chi square value, DF = Degree of freedom

### Validity, reliability and descriptive statistics

**Fig. 2:**
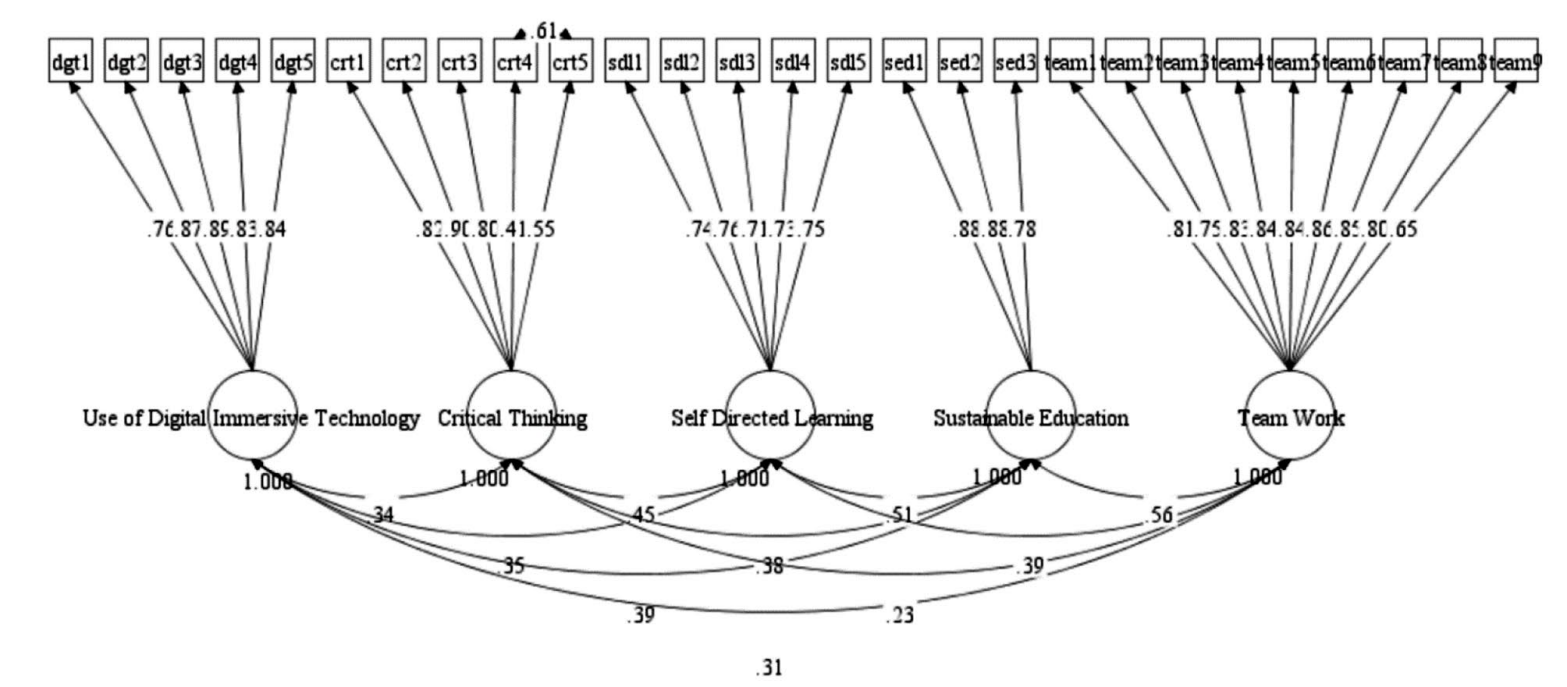
Measurement model

Additionally, the validity and reliability of the model was further confirmed by calculating the convergent and discriminant validity [58–60]. It was important to assess the constructs’ discriminant validity to confirm that the concepts which were not theoretically related were unrelated. For this purpose, we first computed the SQRT (squared root) of AVE (average variance extracted) and compared these values with correlation of the of the constructs. The analysis confirmed that the SQRT of AVE was significantly the greater than their corresponding correlations, thus confirming that the variables which were theoretically different were actually different [61], depicting the existence of discriminant validity. Furthermore, for the assessment of convergent validity, we examined the standardized factor loadings & AVE of the constructs [62]. It was observed that the factor loadings (STDYX) and AVE met the recommended criteria [59–62], because AVE was significantly higher than 0.5 and STDYX of all the factors (see Fig. 2) were greater than 0.4 [63, 64].

In addition to this, the internal consistency of the scale was examined by computing composite reliability (CR). The analysis confirmed (see Table 2) that the CR values of all the observed variables were significantly higher than 0.7. The highest value of CR (0.94) was associated to the moderating variable “TEAM”, whereas its lowest value (0.833) was linked to one of the mediating variable “CRT”. Table 2 also provided insights about the normality of the data by highlighting the MEAN, STD values. Looking at the MEAN and STD values, it can be found out that the normality of the data was not violated. Finally, it was essential to compute multicollinearity to confirm that the constructs are not highly correlated. The analysis revealed that although the variables were significantly correlated but still these values were far lesser than 0.9 and thus do not indicate the issue of multicollinearity [65].

### Testing the CMV/ biasness of responses

The data related to the study variables was collected at a single point of time using the same questionnaire, therefore, it was deemed necessary to test whether the individuals’ responses were influenced by their predispositions or not. For this purpose, a test of CMV (common method variance) was conducted. A famous technique of Harman’s single-factor method was applied to determine the biasness of the respondents if any. The results of the test revealed that one factor only explained 35.09% of the variance. These results that the relationship between the variables did not occur due to the biased beliefs of the respondents because the one factor accounted for significantly lesser than 50% of the variance. It was confirmed that there was no issue of common method biasness [66]. Table 2 presents descriptive statistics, correlation and discriminant validity indices.

**Table 2:** Descriptive statistics, correlation and discriminant validity

Construct	DGIT	CRT	SDL	SED	TEAM
DGIT	**0.824**				
CRT	0.339**	**0.840**			
SDL	0.354**	0.450**	**0.718**		
SED	0.391**	0.376**	0.514**	**0.848**	
TEAM	0.314**	0.228**	0.393**	0.557**	**0.869**
Mean	4.948	4.934	5.641	5.846	6.381
S.D.	1.034	0.722	0.763	0.953	0.811
CR	0.923	0.833	0.857	0.887	0.943
AVE	0.71	0.52	0.54	0.72	0.649

*Noten* = 304, S. D = Standard deviation, DGIT = Use of digital immersive technology, SED = Sustainable education, CRT = Critical thinking, SDL = Self-directed learning, Team = Team work

### Hypothesis testing for direct, indirect and moderated relationships

**Fig. 3:**
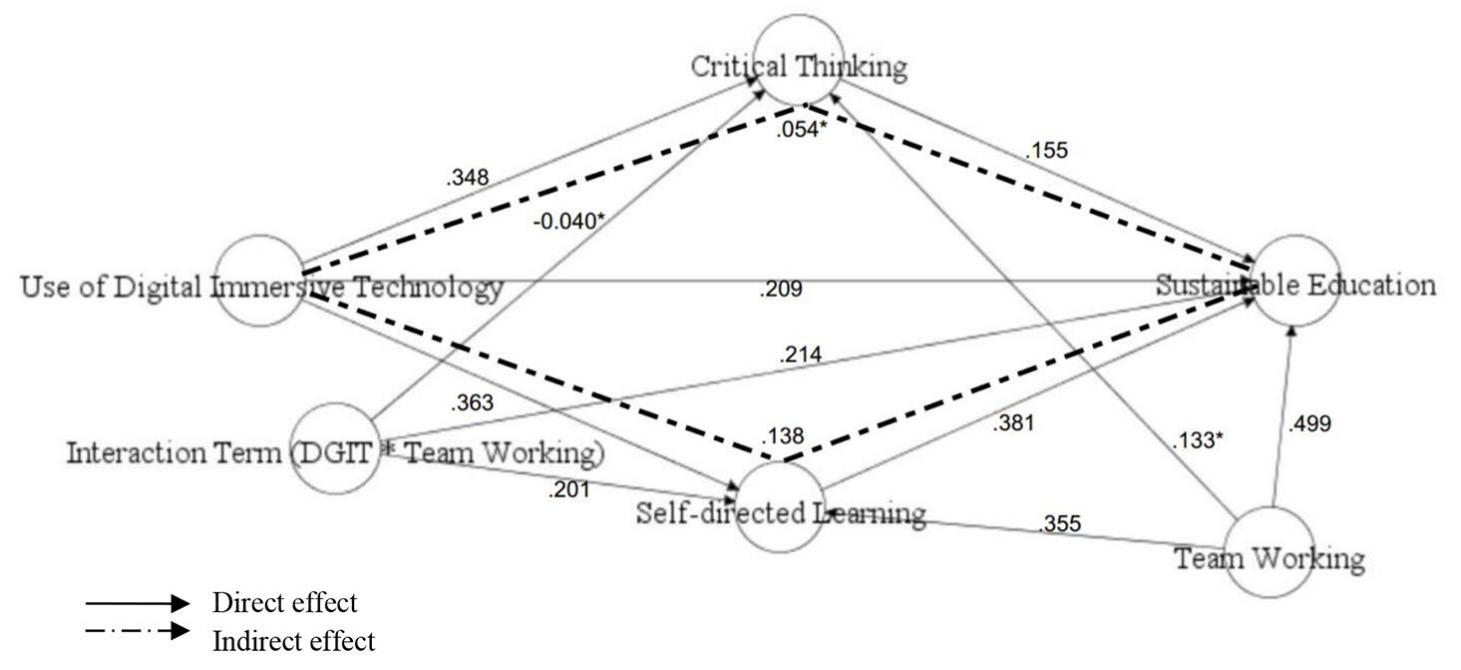
Structural model

Moving ahead with the hypothesis testing, the structural equation modelling approach was applied to run the direct, indirect and the moderated relationships. As the model involved the testing of both direct and indirect associations, therefore, the significance of all such hypothesis was first examined by applying SEM. The first 5 hypothesis were linked to the assessment of direct associations between DGIT, CRT, SDL and SED. The results revealed that DGIT significantly predicted SED, β (STDYX) = 0.209, SE = 0.061, T-value = 3.432, P-value = 0.00, CRT β (STDYX) = 0.348, SE = 0.056, T-value = 6.185, P-value = 0.00, and SDL, β (STDYX) = 0.363, SE = 0.057, T-value = 6.378, P-value = 0.00. Therefore, H1, H2 and H3 were significant and supported.H4 was related to the assessment of direct association between CRT and SED, whereas H5 was linked to the assessment of SDL-SED relationship. As per the expectations, both CRT and SDL positively and significantly predicted SED, β (STDYX) = 0.155, SE = 0.065, T-value = 2.393, P-value = 0.017, β (STDYX) = 0.381, SE = 0.062, T-value = 6.112, P-value = 0.00 respectively. These results (see Table 3; Fig. 3) confirmed all the direct paths in the model were positive and significant.

**Table 3:** Hypothesis testing for direct effects

Hypotheses	β	SE	T-Value	P-Value	Outcomes
H1: DGIT-SED	0.209	0.061	3.432	0.001	Supported
H2: DGIT-CRT	0.348	0.056	6.185	0.000	Supported
H3: DGIT-SDL	0.363	0.057	6.378	0.000	Supported
H4: CRT-SED	0.155	0.065	2.393	0.017	Supported
H5: SDL-SED	0.381	0.062	6.112	0.000	Supported

*Note* β = STDYX, SE = Standard error, DGIT = Use of digital immersive technology, SED = Sustainable education, CRT =

Critical thinking, SDL = Self-directed learning

Combined with the direct effects, we also examined the mediated paths present in the structural model. For this purpose, the mediating role of CRT and SDL was investigated between the independent variable “DGIT” and dependent variable “SED” respectively. The following Table 4 contains detailed information about the indirect effects.

**Table 4:** Hypothesis testing for mediation

Hypotheses	β (SIE)	SE	T-Value	95% CI	P-Value	Outcomes
H6: Mediation of CRT between DGIT & SED	0.054	0.029	1.874	0.007—0.101	0.061	Not Supported
H7: Mediation of SDL between DGIT & SED	0.138	0.034	4.083	0.082—0.194	0.000	Supported

*Note* β = STDYX, SIE = Specific indirect effect, SE = Standard error, CI = Confidence interval, DGIT = Use of digital immersive

technology, SED = Sustainable education, CRT = Critical thinking, SDL = Self-directed learning

The significance of the indirect paths was determined by observing the p-value, t-value, and 95% ULCI and LLCI of the specific indirect effects. It is recommended that the sample should be artificially increased to 1000 bootstraps at least, for obtaining the reliable results of mediations [67] and trust worthy confidence intervals [68, 69]. However, we opted for an even larger no. of bootstraps i.e., 5000-bootsraps. Continuing with the analysis, H6 assumed that CRT will significantly mediate the association of DGIT and SED. Contrary to the expectations, it was proved that this specific indirect effect was insignificant* because of its STDYX β-value = 0.054 (SIE), SE = 0.029, T-value = 1.874 and P-value = 0.061 (see Table 4; Fig. 3). Nevertheless, a test for 95% of CI proved to be significant because its upper and lower values did not pass through zero 0.007—0.101(692). Therefore, being stringent on the measure, it was interpreted that H6 was not supported. Likewise, H7 postulated that SDL will be useful for connecting the DGIT and SED. As per the prediction, this indirect effect was positive and significant, STDYX (β) = 0.138, with SE = 0.034, T-value = 4.083 and P-value was 0.000. This significance was also proved through the examination of 95% CI as the lower limit of 95% and upper limit of 95% CI did not pass through zero i.e., 0.082—0.194. Therefore, H7 was supported (see Table 4; Fig. 3).

Finally, we examined the moderating role of teamwork between DGIT-SED, DGIT-CRT and DGIT-SDL through H8, H9, H10 respectively. It was established that the effect of interaction term (DGIT x TEAM) was significant and positive β = 0.214, SE = 0.090, T-Value = 2.369 and its p-value was 0.018. Additionally, the 95% CI also did not go through zero (see Table 5; Fig. 3). Thus, H8 was supported. As far as the moderating role of TEAM on DGIT-CRT path is concerned, the moderation proved to be negative and highly insignificant* in this case. It can be observed that β= -0.40, SE = 0.096, T-Value = − 0.413 and its p-value was 0.680. In addition to this, the 95% of CI also proved that the moderation was insignificant* (see Table 5; Fig. 3).

**Table 5:** Moderation analysis

Hypotheses	β	SE	T-Value	P-Value	95% CI	Outcomes
H8: Moderating role of TEAM on DGIT-SED relationship	0.214	0.090	2.369	0.018	0.065–0.362	Supported
H9: Moderating role of TEAM on DGIT-CRT relationship	-0.040	0.096	-0.413	0.680	− 0.197–0.118	Not Supported
H10: Moderating role of TEAM on DGIT-SDL relationship	0.201	0.091	2.231	0.027	0.051–0.351	Supported

*Note* β = STDYX, SE = Standard error, CI = Confidence interval, DGIT = Use of digital immersive technology, SED = Sustainable education, CRT = Critical thinking, SDL = Self-directed learning, Team = Team work

At the end, the moderation of TEAM on the DGIT-SDL path was investigated. Looking at results, it was established that the moderating variable “TEAM” significantly strengthened the relationship of DGIT-SDL, i.e., STDYX β-coefficient = 0.201 SE = 0.091, T-value = 2.231, p-value = 0.027. Moreover, the analysis of 95% CI revealed that ULCI and LLCI did not include zero (see Table 5; Fig. 3) indicating that the moderating role was positive and significant. Thus, as per the assumptions of the researcher, H10 was supported.

## Discussion

The findings of the study suggest that digital immersive technology has the potential to promote critical thinking and self-directed learning among English language learners. The use of digital immersive technology creates an immersive environment that enables learners to engage in a more interactive and participatory learning experience. It encourages learners to actively engage in the learning process, take responsibility for their learning, and develop critical thinking skills by providing opportunities to solve problems and make decisions. Moreover, the results suggest that teamwork can moderate the relationship between digital immersive technology and critical thinking and self-directed learning. Specifically, digital immersive technology combined with collaboration can enhance critical thinking and self-directed learning among English language learners. Teamwork allows learners to collaborate, share ideas, and learn from one another, improving their necessary thinking skills and promoting self-directed learning. The study findings have important implications for English language educators and policymakers. Using digital immersive technology in English language education can promote critical thinking and self-directed learning, essential skills for achieving sustainability in education. The study suggests that digital immersive technology should combine with teamwork to enhance its effectiveness in promoting critical thinking and self-directed learning. Therefore, educators should design effective strategies incorporating digital immersive technology and collaboration in English language teaching and learning. With almost 1.5 billion speakers worldwide, English is one of the most frequently spoken languages. It is the language of technology, science, and international commerce, making it a crucial talent for anybody looking to excel in the global economy. In nations where English is the primary language of instruction and communication, English language proficiency can open doors to further education options and job chances. Additionally, learning English can give you access to many knowledge and materials in entertainment, media, and literature. As a result, in today’s linked world, communicating effectively in English is essential for both personal and professional success.

## Conclusion

In recent years, teaching English as a second language using digital immersion technology has become more common. These technological advancements give students a dynamic and exciting learning experience through virtual and augmented reality, 3D simulations, and instructional games. It is still unclear, nevertheless, how using digital immersive technology, collaboration, and critical thinking are related. Problem-solving and individual learning may be more important for developing necessary thinking skills than teamwork. However, digital immersive technology can encourage self-directed learning, collaboration, and critical thinking. Interactive instructional games, for instance, might present difficult situations that call for original thought and problem-solving abilities. Students’ curiosity and enthusiasm for learning through virtual and augmented reality and interactive feedback can help them find areas for improvement. Students’ teamwork and collaboration can be facilitated through digital immersive technology, motivating them to work together towards a common objective. In the end, digital immersion technology has the potential to significantly contribute to the achievement of sustainability in education by giving students the abilities and information required to thrive in the twenty-first century.

## Policy, practical, and theoretical implications

### Policy implication

Regardless of socioeconomic background, policies should guarantee that all pupils have access to digital immersive technology. It can be by financing schools specifically intended for them or by forming alliances with IT firms to provide people with care. Also, policies addressing how to incorporate digital immersive technology into language programs for teachers successfully should be devised. Helping instructors stay current on the newest technologies and best practices can involve ongoing support, resources, and professional development opportunities. Governments should support the creation of a digital immersive language curriculum and offer recommendations on how to apply these technologies best to foster critical thinking and self-directed learning. It may entail creating criteria for measuring language proficiency online and standards for planning and executing immersive language-learning activities. Digital immersive technology should be encouraged by policies to foster student participation and teamwork. It can involve creating language-learning activities that encourage students to cooperate to attain a common objective and allow them to reflect on their learning and share their experiences.

Digital immersion technology has the potential to encourage teamwork, self-directed learning, and critical thinking, all of which are crucial for achieving sustainability in education. Policymakers may assist in ensuring that kids have access to technology by creating policies that encourage teacher training, curriculum creation, and cooperation.

### Practical implications

Students can benefit from tailored learning experiences made possible by digital immersion technology. Students can practice their language abilities in context by using virtual environments that teachers can construct that mimic real-life situations. Forcing children to use their linguistic skills to solve problems can aid in developing their critical thinking abilities. Moreover, collaboration and teamwork among students can be facilitated through digital immersive technology. Students can collaborate on activities, talk about linguistic ideas, and give one another feedback by using digital platforms. Collaborative learning like this promotes independent learning and aids in the growth of crucial interpersonal and collaboration abilities in pupils. Thanks to digital immersion technologies, students may gain access to real-world experiences that they might not otherwise have. For instance, students can tour historical landmarks, engage with English-speaking natives in virtual environments, or visit virtual museums. These opportunities can foster critical thinking abilities in pupils and help them gain a deeper understanding of the language and culture. Students may benefit from digital immersive technology’s freedom in their learning. Online tools and resources for language study are always available to students and from any location. It can assist students in taking ownership of their education and fostering their capacity for independent study. Assessment of student learning can also use digital immersion technologies. Teachers can use digital platforms to monitor student progress, give comments, and pinpoint areas where students need more assistance. It can encourage self-directed learning by providing students with the resources they need.

### Theoretical implications

The constructivist learning philosophy emphasizes active and interactive learning via inquiry and discovery and aligns with digital immersive technology. Thanks to digital immersive technology, students can learn a language in a virtual setting and connect language ideas to practical situations. Having the freedom to create their conceptualizations of the language encourages critical thinking and self-directed learning in the students. Digital immersive technology is fundamentally dependent on collaborative learning. It facilitates student collaboration and communication while supporting the social creation of knowledge. As a result, students gain crucial abilities required for achieving sustainability in education, including problem-solving, critical thinking, and effective communication.

By giving students the resources, they need to keep track of their progress and assume responsibility for their learning, digital immersion technology can also support self-directed learning. It is consistent with the self-directed learning idea, which strongly emphasizes the necessity of giving students ownership over their own learning. The move to technology-enhanced understanding, which acknowledges the importance of technology in augmenting and transforming the learning experience, is represented by digital immersive technology. In addition to encouraging sustainability in education, this strategy acknowledges the potential of digital immersion technology to foster critical thinking, self-directed learning, and teamwork. The development of 21st-century abilities, such as teamwork, critical thinking, and digital literacy, which are crucial for success in the modern world, is encouraged by digital immersion technology. The 21st-century learning theory, which emphasizes the need to prepare students for the demands of the digital age, is in line with this.

### Limitations

Future studies should take the study’s shortcomings into account. Secondly, the study’s findings cannot extrapolate to other fields or subjects because it solely examined the use of digital immersion technology in English language teaching. A mixed-methods approach to research could be employed in the future to provide a more thorough knowledge of the role of digital immersive technology in fostering critical thinking and self-directed learning. The study second used a quantitative research design. Finally, employing digital immersion technology in English language instruction may face obstacles and difficulties not examined in the study. Future studies could explore potential barriers and challenges and create plans to overcome them.

### Electronic supplementary material

Below is the link to the electronic supplementary material.

Supplementary Material 1

## Data Availability

The data will be made available by the authors upon request from the corresponding author.

## References

[1] Dobakhti L (2021). A study of the role of English Language for General, Specific and Academic purposes. Lang Relat Res.

[2] Kapranov O. The discourse of sustainability in English Language Teaching (ELT) at the University of Oxford: analyzing discursive representations. J Teacher Educ Sustain, 24, 1, 3922, pp.35–48. 10.2478/jtes-2022-0004.

[3] Jonassen DH, Rohrer-Murphy L (1999). Activity theory as a framework for designing constructivist learning environments. Education Tech Research Dev.

[4] Schmid R, Pauli C, Petko D. (2022). Examining the use of digital technology in schools with a school-wide approach to personalized learning. Education Tech Research Dev, 1–24.

[5] Haleem A, Javaid M, Qadri MA, Suman R (2022). Understanding the role of digital technologies in education: a review. Sustainable Oper Computers.

[6] Lee H, Hwang Y (2022). Technology-enhanced education through VR-making and metaverse-linking to foster teacher readiness and sustainable learning. Sustainability.

[7] Qureshi MI, Khan N, Raza H, Imran A, Ismail F. (2021). Digital technologies in education 4.0. Does it enhance the effectiveness of learning?.

[8] Hung H-T, Yang JC, Hwang G-J, Chu H-C, Wang C-C (2018). A scoping review of research on digital game-based language learning. Comput Educ.

[9] Bower M, DeWitt D, Lai JWM (2020). Reasons associated with preservice teachers’ intention to use immersive virtual reality in education. Br J Edu Technol.

[10] Araiza-Alba P, Keane T, Chen WS, Kaufman J (2021). Immersive virtual reality as a tool to learn problem-solving skills. Comput Educ.

[11] Alismaiel OA, Cifuentes-Faura J, Al-Rahmi WM. (2022). Social media technologies used for education: An empirical study on TAM model during the COVID-19 pandemic. *Frontiers in Education*, *7*.

[12] Al Hakim VG, Yang S-H, Liyanawatta M, Wang J-H, Chen G-D (2022). Robots in situated learning classrooms with immediate feedback mechanisms to improve students’ learning performance. Comput Educ.

[13] Mallam SC, Nazir S, Renganayagalu SK (2019). Rethinking maritime education, training, and operations in the digital era: applications for emerging immersive technologies. J Mar Sci Eng.

[14] Kitson A, Prpa M, Riecke BE (2018). Immersive interactive technologies for positive change: a scoping review and design considerations. Front Psychol.

[15] Blyth C (2018). Immersive technologies and language learning. Foreign Lang Annals.

[16] Wu L, Wang D, Evans JA (2019). Large teams develop and small teams disrupt science and technology. Nature.

[17] Fia M, Ghasemzadeh K, Paletta A. How higher Education Institutions Walk their talk on the 2030 agenda: a systematic literature review. Higher Education Policy; 2022. 10.1057/s41307-022-00277-x PMC9226290 35765671

[18] Ramadhan S, Sukma E, Indriyani V. (2019). Environmental education and disaster mitigation through language learning. *IOP Conference Series: Earth and Environmental Science*, *314*(1), 12054.

[19] Wang P, Wu P, Wang J, Chi H-L, Wang X (2018). A critical review of the use of virtual reality in construction engineering education and training. Int J Environ Res Public Health.

[20] Radianti J, Majchrzak TA, Fromm J, Wohlgenannt I (2020). A systematic review of immersive virtual reality applications for higher education: design elements, lessons learned, and research agenda. Comput Educ.

[21] Suh A, Prophet J (2018). The state of immersive technology research: a literature analysis. Comput Hum Behav.

[22] Erlam G, Smythe L, Clair W-S, V. Action research and millennials: improving pedagogical approaches to encourage critical thinking. Nurse Education Today; 2018. 10.1016/j.nedt.2017.11.023 29197689

[23] Duncan KJ (2020). Examining the effects of immersive game-based learning on student engagement and the development of collaboration, communication, creativity and critical thinking. TechTrends.

[24] Gómez-Galán J, Vázquez-Cano E, de la Luque A, López-Meneses E (2020). Socio-educational impact of augmented reality (AR) in sustainable learning ecologies: a semantic modeling approach. Sustainability.

[25] Hein RM, Wienrich C, Latoschik ME. (2021). A systematic review of foreign language learning with immersive technologies (2001–2020). AIMS Electron Electr Eng, *5*(2).

[26] Halabi O (2020). Immersive virtual reality to enforce teaching in engineering education. Multimedia Tools Appl.

[27] Lian J, Chai CS, Zheng C, Liang J-C (2021). Modelling the relationship between Chinese university students’ authentic language learning and their English self-efficacy during the COVID-19 pandemic. Asia-Pacific Educ Researcher.

[28] Ho RC, Song BL (2022). Immersive live streaming experience in satisfying the learners’ need for self-directed learning. Interact Technol Smart Educ.

[29] Alam A (2022). Mapping a sustainable future through conceptualization of transformative learning framework, education for sustainable development, critical reflection, and responsible citizenship: an exploration of pedagogies for twenty-first century learning. ECS Trans.

[30] Gatti L, Ulrich M, Seele P (2019). Education for sustainable development through business simulation games: an exploratory study of sustainability gamification and its effects on students’ learning outcomes. J Clean Prod.

[31] Kopnina H (2020). Education for the future? Critical evaluation of education for sustainable development goals. J Environ Educ.

[32] Al-Adwan AS, Nofal M, Akram H, Albelbisi NA, Al-Okaily M. (2022). Towards a Sustainable Adoption of E-Learning Systems: The Role of Self-Directed Learning. *Journal of Information Technology Education: Research*, *21*.

[33] Shafait Z, Yuming Z, Meyer N, Sroka W (2021). Emotional intelligence, knowledge management processes and creative performance: modelling the mediating role of self-directed learning in higher education. Sustainability.

[34] Shafait Z, Khan MA, Bilan Y, Oláh J. (2021). Modeling the mediating roles of self-directed learning and knowledge management processes between emotional intelligence and learning outcomes in higher education. PLoS ONE, 16(7), e0255177. 10.1371/journal.pone.0255177 PMC8315535 34314452

[35] Lee E-Y, Jeon YJJ (2020). The difference of user satisfaction and net benefit of a mobile learning management system according to self-directed learning: an investigation of cyber university students in hospitality. Sustainability.

[36] Jeong K-O (2022). Facilitating sustainable self-directed learning experience with the use of mobile-assisted language learning. Sustainability.

[37] Kocak O, Coban M, Aydin A, Cakmak N (2021). The mediating role of critical thinking and cooperativity in the 21st century skills of higher education students. Think Skills Creativity.

[38] Badilla-Quintana MG, Sepulveda-Valenzuela E, Salazar Arias M (2020). Augmented reality as a sustainable technology to improve academic achievement in students with and without special educational needs. Sustainability.

[39] Ye Q, Zhou R, Anwar MA, Siddiquei AN, Hussain S, Asmi F (2022). Virtual reality-based learning through the lens of eudaemonic factors: reflective thinking as a game changer. Think Skills Creativity.

[40] Park JH, Lee H (2021). The influence of Self-Directed Learning and Learning Commitment on Learning Persistence Intention in Online Learning: Mediating Effect of Learning Motivation. Int J Adv Cult Technol.

[41] Makri A, Vlachopoulos D, Martina RA (2021). Digital escape rooms as innovative pedagogical tools in education: a systematic literature review. Sustainability.

[42] Sandoval Henríquez F, Badilla Quintana MG. (2021). *Students’ Immersive Experience in Initial Teacher Training in a Virtual World to Promote Sustainable Education: Interactivity, Presence, and Flow*.

[43] Lai Y, Saab N, Admiraal W (2022). University students’ use of mobile technology in self-directed language learning: using the integrative model of behavior prediction. Comput Educ.

[44] Whewell E, Caldwell H, Frydenberg M, Andone D. (2022). Changemakers as digital makers: connecting and co-creating. Educ Inform Technol, *27*(5). 10.1007/s10639-022-10892-1 PMC8784248 35095325

[45] Tilak S, Glassman M, Lu M, Wen Z, Pelfrey L, Kuznetcova I, Lin T-J, Anderman EM, Calvit M, Ching K (2023). Investigating social studies teachers’ implementation of an immersive history curricular unit as a cybernetic zone of proximal development. Cogent Educ.

[46] Hwang G, Chang C, Chien S. (2022). A motivational model-based virtual reality approach to prompting learners’ sense of presence, learning achievements, and higher‐order thinking in professional safety training. Br J Edu Technol, *53*(5).

[47] Yusuf F, Ali A (2022). Exploring students’ perception on using live worksheet as self-directed learning of listening skills in Online Education. Utamax: J Ultimate Res Trends Educ.

[48] Osborne J. (2008). *Best practices in quantitative methods*. 10.4135/9781412995627.

[49] He Q, Zhang Y (2023). Residential locations and residential moves between the city centre and suburb in Beijing, China. Habitat Int.

[50] Kothari. (2004). *Research Methodology: Methods and Techniques - C. R. Kothari - Google Books*.

[51] Al-Sharafi MA, Al-Emran M, Iranmanesh M, Al-Qaysi N, Iahad NA, Arpaci I. (2022). Understanding the impact of knowledge management factors on the sustainable use of AI-based chatbots for educational purposes using a hybrid SEM-ANN approach. 10.1080/10494820.2022.2075014

[52] Duncan T, Mckeachie WJ. (2016). *Available from: Teresa Duncan Retrieved on*. 12.

[53] Ahmed W. Understanding self-directed learning behavior towards digital competence among business research students: SEM-neural analysis. Educ Inform Technol. 2022;1–30. 10.1007/S10639-022-11384-Y/TABLES/8.

[54] Gheorghe A, Oana, ·, Fodor C, Petru, ·, Curșeu L, Trif S, Cirebea L (2022). The effect of humor and perceived social interdependence on teamwork engagement in student groups. Curr Psychol 2022.

[55] Sakaria D, Maat SM, Matore MEEM (2023). Examining the optimal choice of SEM Statistical Software packages for sustainable Mathematics Education: a systematic review. Sustain 2023.

[56] *New developments in Mplus version 7: Part 2*. Presentation at Utrecht University. Retrieved from https://www. statmodel &#8230.

[57] Geiser C. (2022). *Handbook of Structural Equation Modeling - Google Books*.

[58] Fornell C, Larcker D (1981). Evaluating Structural equation models with unobservable variables and measurement error. J Mark Res.

[59] Thien LM. (2019). Assessing a second-order quality of school life construct using partial least squares structural equation modelling approach, 43(3), 243–256. 10.1080/1743727X.2019.1662779

[60] Fornell C, Larcker DF (1981). Structural equation models with unobservable variables and measurement error: Algebra and statistics.

[61] Whittaker TA. (2011). A Beginner’s Guide to Structural Equation Modeling (3rd ed.), 18(4), 694–701. 10.1080/10705511.2011.607726

[62] Suárez-Albanchez J, Blazquez-Resino J, Gutierrez-Broncano J, Jimenez-Estevez S, Queirós P, Ruiz PS, Yañez-Araque B (2021). Occupational Health and Safety, Organisational Commitment, and turnover intention in the Spanish IT Consultancy Sector. Int J Environ Res Public Health 2021.

[63] Hair J (2009). Multivariate Data Analysis: a global perspective.

[64] Memon AH, Rahman IA. SEM-PLS analysis of inhibiting factors of cost performance for large construction projects in Malaysia: perspective of clients and consultants. Sci World J. 2014;2014. 10.1155/2014/165158. 10.1155/2014/165158 PMC3947800 24693227

[65] Midi H, Sarkar SK, Rana S, Midi H, Rana S. Collinearity diagnostics of binary logistic regression model. J Interdisciplinary Math. 2010;13(3):253–67. 10.1080/09720502.2010.10700699.

[66] Saris WE, Gallhofer IN. (2014). *Design, Evaluation, and Analysis of Questionnaires for Survey Research Wiley Series in Survey Methodology*. 1–377.

[67] Cui C, Raslan R, Korolija I, Chalabi Z. On the robustness of thermal comfort against uncertain future climate: a bayesian bootstrap method. Build Environ. 2022;109665. 10.1016/J.BUILDENV.2022.109665.

[68] Mackinnon D. Introduction to statistical mediation analysis. Introduction Stat Mediation Anal. 2012;1–477. 10.4324/9780203809556/INTRODUCTION-STATISTICAL-MEDIATION-ANALYSIS-DAVID-MACKINNON.

[69] Shrout PE, Bolger N (2002). Mediation in experimental and nonexperimental studies: new procedures and recommendations. Psychol Methods.

