# Supplementary material for: Understanding the role of digital immersive technology in educating the students of english language: does it promote critical thinking and self-directed learning for achieving sustainability in education with the help of teamwork?
Source: BMC Psychol. 2024 Mar 13;12:144. doi: 10.1186/s40359-024-01636-6 (PMC10938835; doi:10.1186/s40359-024-01636-6)
Supplement: Supplementary file 1 — Supplementary Material 1 [file 40359_2024_1636_MOESM1_ESM.docx]

Dear participant

Please specify your idea about each of the following items, using the following options:

1= Strongly disagree 2= disagree 3= somehow agree, 4=Agree 5= Strongly agree

Use of Digital Immersive Technology (Modified Items - 3 items):

1. I effectively integrate digital immersive technology into my educational experiences.
2. Digital immersive technology enhances my understanding and engagement in learning activities.
3. I frequently utilize digital immersive technology to explore and experiment with educational content.

**Self-Directed Learning (Modified Items - 9 items):**

1. I take the initiative to set my own learning goals.
2. I actively seek out resources and materials to support my learning.
3. I can manage my time effectively when engaging in self-directed learning.
4. Self-directed learning enhances my sense of autonomy and independence.
5. I reflect on my learning experiences and adjust my approach accordingly.
6. I enjoy the process of self-directed learning and find it motivating.
7. I consistently follow through on my self-directed learning plans.
8. I am confident in my ability to learn independently.
9. I regularly assess the effectiveness of my self-directed learning strategies.

**Sustainable Education (Modified Items):**

1. I am aware of the importance of sustainability in education.
2. I actively seek out sustainability-focused learning opportunities.
3. I believe that sustainable education plays a crucial role in addressing global challenges.
4. I incorporate sustainable principles into my academic work.
5. I am committed to promoting sustainability in my educational pursuits.

**Critical Thinking (Modified Items):**

1. I can critically analyze and evaluate complex issues.
2. I regularly question assumptions and seek evidence to support my ideas.
3. Critical thinking helps me make well-informed decisions.
4. I can identify and challenge biases in my thinking.
5. I value open-mindedness and a willingness to consider alternative viewpoints.

**Team Working (Modified Items):**

1. I collaborate effectively with team members to achieve common goals.
2. I am skilled at communicating and sharing ideas within a team.
3. I can adapt to different roles and responsibilities in a team setting.
4. I value diversity and inclusivity in team interactions.
5. I believe that teamwork enhances the quality of work and outcomes.
